# Living After Pelvic Exenteration: A Mixed-Methods Synthesis of Quality-of-Life Outcomes and Patient Perspectives

**DOI:** 10.3390/jcm14186541

**Published:** 2025-09-17

**Authors:** Vlad Rotaru, Elena Chitoran, Aisa Gelal, Giuseppe Gullo, Daniela-Cristina Stefan, Laurentiu Simion

**Affiliations:** 1 Medicine School, “Carol Davila” University of Medicine and Pharmacy, 050474 Bucharest, Romania; 2 General Surgery and Surgical Oncology Department I, Bucharest Institute of Oncology, “Prof. Dr. Alexandru Trestioreanu”, 022328 Bucharest, Romania; 3 Department of Obstetrics and Gynecology, Villa Sofia Cervello Hospital, University of Palermo, 90146 Palermo, Italy; 4 Department of Human, Biological and Translational Medical Sciences, University of Namibia, Windhoek 10026, Namibia

**Keywords:** pelvic exenteration, quality of life, body image, sexual health, mixed methods, qualitative interviews, survivorship, psycho-oncology, patient-reported outcomes

## Abstract

**Background/Objective**: Pelvic exenteration (PE) is a radical procedure with significant physical and psychosocial consequences. Despite increasing survival rate following PE, quality of life (QoL) outcomes remain inconsistently reported and poorly understood in clinical practice. This study aims to explore: (1) What is the current evidence on QoL after pelvic exenterations? and (2) How do patient-reported experiences align with or differ from findings in the literature? **Methods**: We conducted a mixed-methods study consisting of two components: (1) a qualitative analysis of 5 in-depth, semi-structured interviews with patients who underwent PE for advanced pelvic cancers; and (2) a narrative review of 28 quantitative and qualitative studies evaluating QoL after PE, published between 1975 and 2023, encompassing 1149 patients. Thematic analysis was performed using an interpretative phenomenological approach. **Results**: Qualitative findings revealed recurrent themes of identity disruption, social withdrawal, emotional resilience, and a need for personalized, preoperative information. Patients often described a mismatch between surgical expectations and lived experience, and expressed a strong desire for better psychological support and realistic communication. The review of published studies showed heterogeneous findings, with moderate recovery in global QoL scores by 6–12 months postoperatively, but persistent impairments in physical function, sexual health, and emotional well-being. Only a minority of studies included patient-reported outcomes tailored to specific domains such as body image or psychological adaptation (17.8%). **Conclusions**: Recovery after pelvic exenteration extends beyond physical healing and requires attention to emotional, social, and existential dimensions. Integrating psycho-oncologic support and patient-centered communication into standard care is essential. This hybrid analysis underscores the importance of addressing quality of life proactively—not only as an outcome, but as a fundamental component of survivorship care.

## 1. Introduction

Pelvic exenteration is among the most radical surgical procedures performed in oncologic practice, entailing en bloc removal of multiple pelvic organs. Initially conceived by Brunschwig in 1948 as a palliative operation, it is now considered a potentially curative intervention in selected cases of locally advanced or recurrent pelvic malignancies, particularly gynecological, colorectal, or urologic cancers [1,2,3]. While it remains a complex and high-risk surgery, advances in imaging, neoadjuvant therapies, reconstructive techniques, and perioperative management have significantly improved patient outcomes over the past two decades [4].

The increasing efficacy of systemic treatments and multimodal oncologic strategies has led to improved survival even in previously deemed inoperable cases. Five-year overall survival following pelvic exenteration now approaches 40–60% in high-volume centers, with particularly favorable outcomes in patients undergoing complete (R0) resections [5,6,7]. As a result, clinicians are faced not only with the challenge of achieving local control, but also with ensuring an acceptable quality of life (QoL) for a growing number of long-term survivors [8,9,10,11,12].

Despite this shift in the survivorship landscape, the attention given to quality-of-life considerations often lags behind. Most QoL data remain limited to structured instruments such as the EORTC QLQ-C30, SF-36, or FACT-C, which provide a snapshot of functional domains but rarely capture the full complexity of the lived experience [13,14]. Moreover, while studies show that some degree of recovery is possible within 6 to 12 months postoperatively, issues related to body image, sexual function, psychological adaptation, and social reintegration remain prevalent and insufficiently explored [15,16].

A major contributing factor is the limited integration of psycho-oncological perspectives into routine preoperative care. Discussions with patients often center around technical feasibility and oncologic indications, while the emotional, relational, and existential consequences of pelvic exenteration are left largely unaddressed. Several studies pointed out persistent deficiencies in preoperative communication and patient involvement in decision-making, especially when confronting complex and life-altering procedures such as pelvic exenterations. Many patients enter surgery without a clear understanding of what the operation will mean for their body, identity, and everyday life [17,18,19,20]. Physicians themselves may feel unequipped or reluctant to raise these topics, and psycho-oncology services—where they exist—are inconsistently integrated into surgical oncology workflows [21,22,23]. The informed consent process often fails to adequately address long-term psychosocial outcomes, leading to patient distress, decisional regret, and unrealistic expectations. This communication gap may result in unmet expectations, psychological distress, and delayed adjustment during recovery [24,25,26,27,28,29].

Qualitative studies have begun to shed light on these hidden aspects of survivor-ship. Through in-depth interviews, researchers have identified themes of social isolation, loss of femininity or masculinity, difficulty in intimate relationships, and stigma associated with stomas or reconstructive changes [15,30,31]. Yet such insights remain fragmented across disciplines and rarely inform the structure of postoperative care or shared decision-making. Existing studies often focus narrowly on physical recovery or stoma management, overlooking broader domains like psychosocial adjustment, sexual function, or long-term emotional well-being.

This study aims to bridge this gap through a hybrid design combining a narrative synthesis of 28 quantitative and qualitative studies with an interpretative analysis of patient interviews. By integrating both measurable outcomes and experiential dimensions, we seek to provide a more nuanced understanding of quality of life after pelvic exenteration and highlight the need for more comprehensive, patient-centered communication and support strategies in oncologic surgery [25,26,27,28,29]. This study aims to explore: (1) What is the current evidence on QoL after pelvic exenterations? and (2) How do patient-reported experiences align with or differ from findings in the literature?

## 2. Materials and Methods

### 2.1. Qualitative Component—Semi-Structured Interviews

This study was conducted in accordance with the Declaration of Helsinki and approved by the Institutional Ethics Committee of Bucharest Institute of Oncology “Prof. Dr. Alexandru Trestioreanu” (IOB)—Protocol Number 24856/24 November 2022. Subjects were selected from the patients who had undergone a form of pelvectomy in IOB. Participation was voluntary and patients who agreed to participate signed an informed consent for participation in this study.

To assess the aspects related to the quality of life (QoL) of the patients after, we conducted a series of semi-structured interviews with 5 patients who received anterior, posterior, or total pelvic exenterations as part of treatment for loco-regionally advanced neoplastic diseases or for various associated complications (such as complex fistulas, hemorrhages, septic complications) willing and able to participate in interviews. Eligible participants were adults, cognitively capable, fluent in Romanian (the language used for the interviews), and at least high-school educated. The interviews were conducted between 12 and 24 months after the intervention—interval selected to allow a stabilized perception of the physical and psychosocial consequences of the intervention, without exceeding the temporal threshold at which memory distortions could influence responses (positive recall bias). The interviews followed a flexible format, with questions adapted in real time to relevant themes raised by patients, to allow for the free expression of personal experiences and to obtain authentic data on self-reported outcomes (PROs), crucial in understanding QoL in these patients [32,33]. A rigid question guide was not used, precisely so as not to restrict the spontaneity of the responses. Interview topics included physical recovery, body image, sexuality, emotional coping, social relationships, and interactions with healthcare professionals.

### 2.2. Narrative Component—Literature Review

The analysis of the responses obtained from patients was complimented by a comprehensive evaluation of the international literature, aiming to identify possible changes in the quality of life in patients who underwent pelvic exenterations using the same 5 criteria mentioned above. We searched four international databases (Medline, PubMed, Scopus, and Embase) for articles available at least as an abstract in English, containing data on the subjects’ quality of life. Additional articles of interest were identified by reference screening. The search included the time frame from data-base inception to June 2025 and the search syntax included terms describing the surgical intervention (pelvic exenteration, exenteration, pelvectomy) and the outcomes of interest (health-related quality of life, quality of life, QoL, questionnaire, body image, physical distress, psychological distress, physical function, psychosexual function, functional outcomes) connected by boolean operators. Studies were included regardless of the type of pathology for which the procedure was indicated, as the changes in QoL are inherent to the surgical method rather than the underlying pathology. We included both quantitative and qualitative studies that reported different medical aspects of QoL, including symptom control and the functional impact of the palliative or curative intervention. Studies that did not contain outcomes of interest were excluded. We also excluded various types of manuscripts like case reports, systematic or narrative reviews, comments, or letters to the editor. Although our review does not want to present itself as a systematic review, but rather as a narrative one in support of our clinical findings, the academic rigors for systematic reviews were met. Our study contains clear search parameters, targeted outcomes, inclusion and exclusion criteria, a clear search flow and identification of relevant studies, evaluation of bias risk, and detailed characteristics for each included study. The Newcastle–Ottawa Scale (NOS) was used for quantitative studies and the Critical Appraisal Skills Programme Checklist (CASP) for qualitative studies. All these aspects are presented in the Result section. Unfortunately, due to heterogeneity in instruments used and reporting systems between studies, a true meta-analytic study was not possible, and we were unable to calculate pooled statistics.

## 3. Results

### 3.1. Qualitative Component—Semi-Structured Interviews

A total of five patients were included in the qualitative analysis. All had locally advanced pelvic cancers, staged between IIIA and IVA. Cases staged as IVA presented with locally invasive tumors (T4a) infiltrating adjacent pelvic organs, without distant metastases (M0). Stage III patients had complex genito-urinary or recto-vaginal fistulas, likely secondary to prior pelvic radiotherapy, which contributed to the indication for pelvic exenteration. Among the patients interviewed, we included two patients with advanced cervical cancers who presented with complex fistulas between the pelvic organs, one who presented with heavy bleeding and a superinfected malignant vaginal tumor, one patient with locally advanced bladder cancer but with minimal symptoms (hematuria), and one patient with a locally occlusive invasive rectal tumor. In all cases, pelvic exenteration was limited to pelvic structures, without the need for resection of extrapelvic organs or major neurovascular structures.

The interviewed group consisted of 3 women and 2 men, aged between 47 and 62 years, and included both scheduled and emergency pelvectomies, total pelvectomies, and hemipelvectomies, as well as a varied number of stomas per patient. Anatomic reconstruction of the digestive/urinary tracts was made whenever technically feasible—as a result, one of the interviewed patients had no stomas. The rest had either colostomy, ureterostomy, or both. Time since surgery varied from 14 to 23 months.

We chose to analyze the quality of life of patients after pelvic exenterations using five characteristics: self-image, social impact (social interactions, sexual function, family relationships, recreational and professional activities), treatment expectations, symptom control, and psychological impact. All patients provided their experiences regarding the procedure, the psychological impact of the diagnostic and procedure, the social and psychological aftermath, as free-form answers which will be discussed at a later point.

### 3.2. Narrative Component—Literature Review

**Figure 1:**
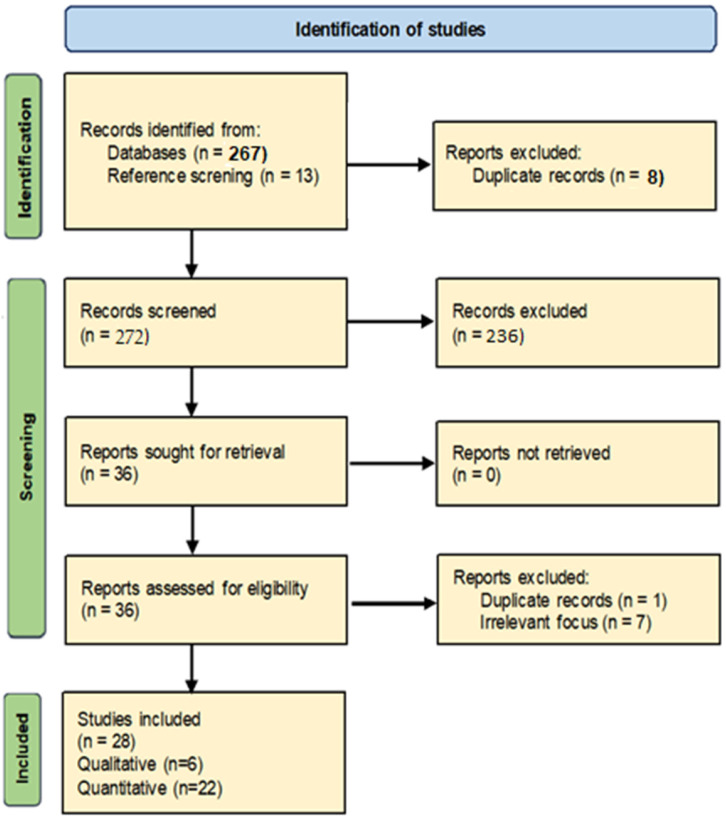
Search flow diagram.

The initial search identified 280 potential relevant articles. After eliminating duplicates and excluding inappropriate studies by title and abstract screening, 36 articles were evaluated as full text, and finally, 28 were included in this narrative literature review (22 quantitative and 6 qualitative). The search flow diagram is presented in Figure 1.

Table 1 summarizes the main characteristics of the included studies.

The studies included encompassed 1814 patients who underwent pelvic exenterations for various malignant diseases, including 446 (24.59%) gynecological cancers, 473 (26.07%) colorectal cancers, and 9 (0.50%) urological cancers. For almost half of the included patients, the origin of the pelvic cancers was unspecified by the authors (48.84%).

**Figure 2:**
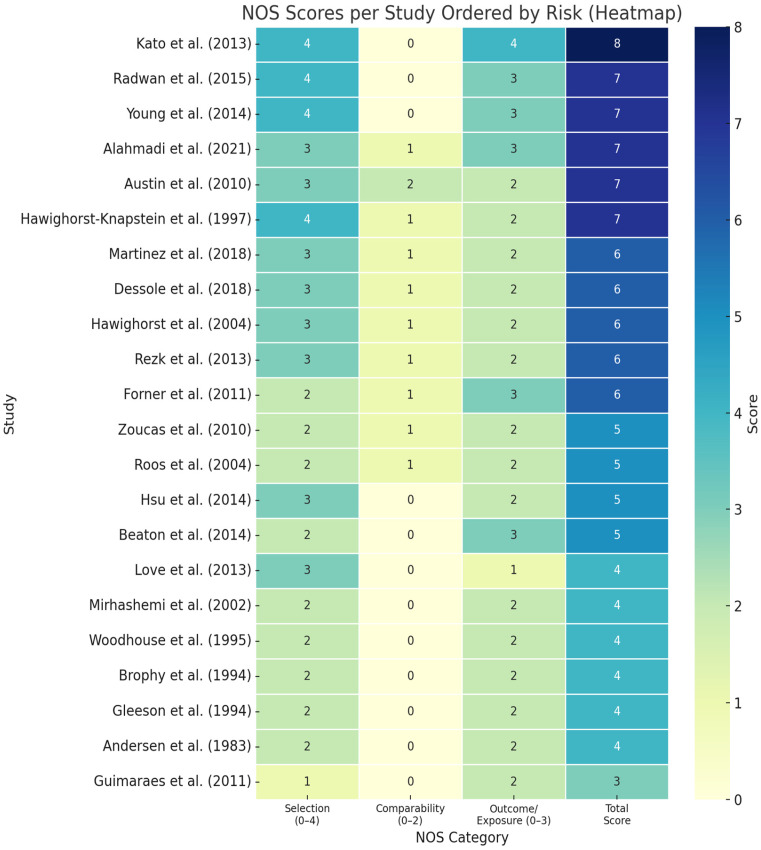
Risk assessment of quantitative studies (Newcastle–Ottawa scale) [2,3,5,6,7,13,14,21,30,31,38,39,40,41,42,43,44,45,46,47,48,49].

**Figure 3:**
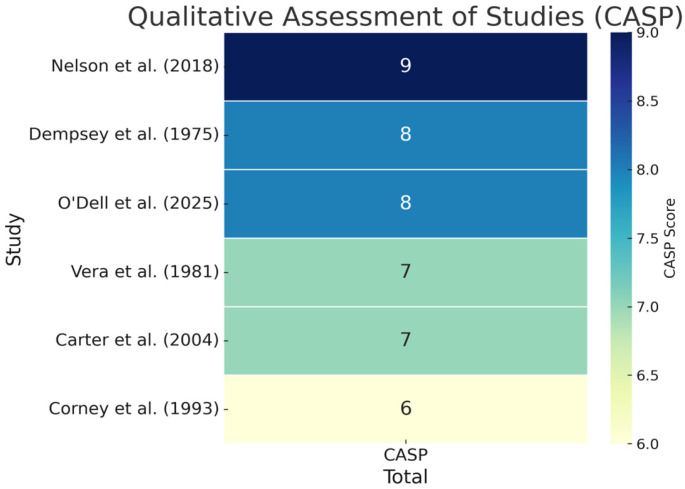
Risk assessment of qualitative studies (Critical Appraisal Skills Programme Checklist—CAPS) [15,16,34,35,36,37].

The quantitative studies were assessed for quality according to the Newcastle–Ottawa Scale (NOS) [50]. Twenty-one studies scored more than 4 points, placing them in the high/intermediate quality group, and 7 were considered high risk. The qualitative studies were assessed according to the Critical Appraisal Skills Programme Checklist (CAPS) [51], with compliance rates of 60–90%. Figure 2 and Figure 3 present the risk evaluation for quantitative and qualitative studies.

The risk of bias varied across included studies. While half of the quantitative articles scored between 6 and 8 on the Newcastle–Ottawa Scale, several had unclear selection of subjects and/or outcome reporting. For qualitative studies, issues like insufficient reflexivity or limited contextual detail were noted. These biases may reduce confidence in the aggregated findings, particularly for underexplored domains such as sexuality or long-term psychosocial adjustments.

## 4. Discussion

Thematic analysis of patient interviews revealed a multidimensional process of psychological adjustment after the surgery, structured around three interrelated domains:

- Bodily disruption and the experience of vulnerability: The physical transformation caused by the exenteration, particularly the presence of stomas and altered pelvic anatomy, was often perceived as a “second illness” distinct from the cancer. Patients often used powerful body metaphors to describe their post-operative bodies, calling themselves “*cut*”, “*mutilated*”, or “*artificial*”. This disruption was deeply connected to a sense of loss of autonomy and control over basic functions. As one participant put it: *“It wasn’t just the illness that left me powerless. It was waking up with holes, tubes, and smells that weren’t mine.”*.
- Rethinking identity and relational isolation: For many women, the loss of their pelvic organs symbolized not only a physical trauma but also a disruption of sexual identity. The absence of vaginal function or the presence of a colostomy was often associated with feelings of shame, desexualization, or self-alienation. Participants described a reduction in their social and relational world, often characterized by fear of intimacy and withdrawal from partners. However, in some cases, supportive relationships became sources of resistance. One participant said, *“My wife is my support, she has always been there for me…”*.
- Meaning-making and post-traumatic reorientation: Despite the invasive nature of the surgery, many patients described a gradual process of adaptation and even personal growth. This transition often involved a reassessment of life priorities, spiritual reflection, and a redefinition of survival over physical integrity. For some, the experience fostered a new sense of empowerment. *“I never thought I could go through this and still laugh. But here I am.”* said one patient. Those who regained independence in daily activities, such as stoma management or mobility, often reported a renewed sense of dignity and purpose.

Findings from literature and our own interviews converged on the importance of sexual function, body image, and social reintegration. However, patient narratives provided richer context on emotional distress and coping, often underexplored in previous work. These findings highlight the complex and nonlinear trajectory of psychological recovery after pelvic exenteration [52]. Rather than a simple return to preoperative baseline, adjustment involved profound changes in self-perception, relationship patterns, and outlook on life. Integrating these perspectives into perioperative and survivorship counseling may improve not only clinical outcomes but also the lived experience of those undergoing one of the most radical interventions in surgical oncology [53,54,55]. These results are compatible with existing literature. Our combined analysis—qualitative and quantitative—suggests that pelvic exenteration, despite its mutilating nature, may be compatible with an acceptable or even good perceived quality of life for many patients, especially in the context of the absence of disease and previous symptomatology. Prospective studies employing validated instruments (e.g., EORTC QLQ-C30, QLQ-CR38, QLQ-CX24, CARES) showed an initial worse QoL, but with significant improvements at 6–12 months post-procedural, especially in the area of global functionality, pain management, and physical activity. For example, in the Martinez et al. study [21], global QoL scores improved significantly between 3 and 12 months post-pelvectomy, especially among patients without major complications and without tumor recurrence. Similarly, in the large cohort analyzed by Alahmadi et al. [6], older patients reported better QoL than younger patients, despite a shorter survival, suggesting better psychosocial adaptation. Qualitative analyses [15,16] highlighted complex coping and re-meaning strategies, in which the absence of chronic pain and the feeling of “rescue by surgery” become essential landmarks in the reconstruction of postoperative identity. In particular, women who undergo anterior pelvectomy (with preservation of the perineum and sometimes the vagina) report a more rapid return to function and body image, in contrast to those with total exenteration. The impact on sexuality and body image is consistently reported to be negative immediately postoperatively, but tends to improve in the long term in patients with psychological support or supportive partners. The classic study by Andersen et al. [31] and more recent investigations (Mirhashemi et al. [45], Corney et al. [36]) support the idea that surgical intervention on the sexual organs profoundly affects not only sexual activity, but also feelings of femininity and intimacy.

### 4.1. Self-Image

Four of our patients reported altered self-image perception, all four having at least one stoma after pelvectomy. The only patient who did not report such changes in self-image was a patient who underwent neobladder reconstruction. Patients who required a digestive stoma and/or a cutaneous ureterostomy were surprised by the extent of the postoperative changes, despite both stating that they had been informed about them by their treating physician prior to surgery.

*Patient 1: “Of course I knew I would wake up with two stoma bags, but seeing myself like this was shocking for me. It took me a long time to get used to it…even now I can’t look at myself properly in the mirror…”.*

*Patient 2: “I changed my clothing style so that it wouldn’t be visible…I started wearing looser, darker clothes.”*

*Patient 3: “I don’t regret the surgery…I had no other choice…but I felt mutilated. I am happy that I don’t have pain anymore, that I don’t bleed anymore, that I don’t smell anymore…but I’ve realized that I would live like this for the rest of my life…”.*

These observations are similar to those in the literature. Four studies, including 133 women who underwent a pelvectomy for mostly gynecological cancers, reported data on postoperative changes in self-image perception by answering various questionnaires (Stauss and Appelt Body Image and EORTC QLQ-CR38/EORTC QLQ-OV28) [14,42,46,56]. These studies showed an initial deterioration of body-image perception, with a gradual return to base values at around 12 months following surgery.

Only a few studies [2,13] have differentiated patients according to the type of urinary diversion or vaginal reconstruction, highlighting differential effects on QoL scores. For example, the use of a VRAM flap for vaginal reconstruction was associated with better body image and more positive perceptions of sexuality [41,45].

The number of stomas was associated with increased body insecurity/discomfort. Patients experienced a lack of attractiveness and decreased self-confidence, feelings that were more pronounced in patients with multiple stomas. Age does not seem to significantly influence the self-image perception [14].

The self-image perception of male patients could not be assessed very well, as all studies conducted on the subject to date have had a sample composed exclusively of women [44,56]. Similarly, studies conducted on the impact of pelvectomy on sexual function have been conducted on female patients [30,31,36,41,45]. The discrepancy between the abundance of information we have on women and the paucity of information on men leads us to conclude that we do not currently know exactly what the repercussions of exenterations are on male patients. The data in the literature should be evaluated objectively and interpreted with caution due to this potential sampling bias.

### 4.2. Social Impact (Social Interactions, Sexual Function, Family Relationships, Recreational and Professional Activities)

Most studies that have evaluated this aspect, such as the study by Rezk et al. [42], have shown a decrease in functional scores in the immediate postoperative period with a gradual return to baseline levels at one year. The first study of this type was that of Demsey in 1970 [34], being a study that included 17 women operated on for gynecological neoplasias who responded to a qualitative interview. The respondents reported a resumption of preoperative social and recreational activities, even if in a modified form, more appropriate to the new realities. No changes in social interactions or interpersonal relationships were described in the postoperative period [34]. Andersen [31] reported similar results. Furthermore, he correlated a lower level of psychological distress in patients who maintained their previous social relationships.

Similarly, the patients interviewed described changes in social activities, but also the period of adaptation to the new situation with the gradual resumption, even in a modified form, of these activities.

*Patient 3: “I was afraid to play with my grandchildren because the stoma pouch might come off…I avoided them for a while…the little ones…I felt guilty but the fear was great. But as I learned to handle the pouch better, I was able to come back to them…last week I went to my niece’s party”.*

Regarding the patients’ couple relationship and marital status, these are profoundly altered by exenterative surgery—some patients reporting abandonment by their husband/partner upon learning of the diagnosis (Vera et al. [35]). However, most spouses/partners expressed initial support, playing a supportive role throughout therapy [34]. The impact of pelvic exenterations on marital status was assessed by Corney et al. [36]—this study concluded that although almost half of the respondents reported sexual dysfunction and deterioration in their sex lives, all women reported that their marriage had improved or remained the same. Although some studies suggest a deterioration in marital status postoperatively, Andersen et al. [31] suggest that this deterioration returns to a baseline level (similar to couples without marital distress) 5 years postoperatively.

Our patients reported varying degrees of couple relationships, ranging from improved relationships with their partner to abandonment.

*Patient 1: “I was depressed for a long time…I only left the house to go to the doctor. It didn’t help that after about a year my husband left… he got tired…of going to the doctor, of the stoma, of me…”.*

*Patient 5: “My wife is my support, she has always been there for me…”.*

Pelvic exenterations can impact marital status through changes in self-image, changes in sexual function, and the inconvenience of stoma wear. The number of stomas is directly correlated with the impact on marital status—patients with two stomas are more affected than those with one stoma, and these are more affected than those without any stoma [46]. The impact on marital status is more profound in young patients [14].

The studies that have investigated the impact of exenterations on sexual function, using a variety of questionnaires, have varying and often conflicting results. These studies reported a decrease in the number of sexually active participants (from 50–100% preoperatively to 12.5–18% postoperatively) [34,35,36,41]. Only one study reported higher rates postoperatively [45]. Two studies [42,45] reported satisfactory postoperative sexual functions and Rezk suggests that if there is a decrease in this function, it returns to baseline values 1 year after surgery.

In addition to psychological discomfort (probably related to altered self-image and decreased confidence)—common to both sexes, women and men report different aspects that affect their sexual activity. Men in the study group both reported decreased libido and erectile dysfunction. Women, on the other hand, face disorders related to dyspareunia (short vagina syndrome) or hormonal disorders (surgically induced menopause).

*Patient 4: “After the surgery I couldn’t anymore…I didn’t expect it to be like this.”*

*Patient 2: “I wasn’t menopausal before…The hot flashes…and the hormonal changes…I didn’t need it anymore, i didn’t want it beacuse it hurt me so much”*

*Patient 3: “When you don’t have a vagina anymore, sex is completely out of the question. Luckily we were already quite old and weren’t that active anymore…we have a different kind of intimacy now, which isn’t necessarily about sex, but which is just as important, if not more so…Not feeling alone is a blessing.”*

Four studies evaluated the possible reasons for sexual dysfunction after exenterations—loss of sexual appetite, persistent vaginal pain, vaginal bleeding or dryness, dysfunctions of the neovagina, and distress caused by the presence of stomas [34,35,36,41]. Nearly half of women reported moderate to severe emotional distress caused by sexual dysfunction [36].

Role changes and increased dependence on others can also affect family life or relationships in the patient’s extended social network. Patients describe shame or fear of rejection from others [37] or feelings of guilt/worry about becoming a burden to their families.

*Patient 5: “Before, I liked going out with friends, walking with my husband in the park… After the operation, I avoided doing these things for a long time…it seemed to me that people were pointing fingers at me. Worse, I thought that the bag would come off and I would make a fool of myself…I had even had nightmares about it. It was only after a year or so that I managed to find the courage to go out into the world again. But I’m still avoiding going in places where I know I don’t have easy access to a bathroom.”*

*Patient 5: “If it weren’t for my wife, I don’t know what I would have done. I didn’t even know how to change the stoma bags properly. I tortured her a lot this year…”.*

In the context of a relatively long postoperative recovery and diminished physical and mental functions, pelvic exenterations have obvious implications for the professional stability and income of patients. Vera et al. [35] report that only one of the 11 patients interviewed was able to return to work as before surgery. The reasons were physical disabilities and limitations imposed by surgical aspects (such as stoma care). Although some patients found other, less demanding jobs, the study group recorded a 53% decrease in earnings compared to preoperative values. The oldest study, Dempsey et al. [34], reported a return to work rate of 80% and that all subjects were able to resume a form of professional activity with reduced responsibilities.

All patients who worked before the operation retired due to illness and reported a decrease in income and a degree of financial difficulty. For patients who have an adequate socio-familial support system, these consequences are easier to bear. On the other hand, in the absence of an optimal support system, the decrease in income and the increase in the degree of dependence can generate a new cause of depression and anxiety.

*Patient 1: “I retired. I couldn’t work anymore. After my husband left, it was just me and my mother… instead of me helping her, the poor woman had to bring me to the doctor. I couldn’t come every time… we live far away”.*

*Patient 2: “I didn’t work anymore after… I felt bad. Luckily my family helped me and we were able to compensate for the decrease in income”.*

*Patient 4: “I was retired before the surgery, but the costs of transportation, coming to the hospital, and the investigations are high…”.*

Another significant aspect when analyzing the social impact of pelvectomies is the difference between older and newer studies reflecting the paradigm shift of women’s role in society. Almost half of all qualitative studies on QoL were conducted in the 1970s and 1980s and were structured around changes more specific to that period (spouse’s reaction to the need for surgery, mood changes and coping strategies, occupational recovery, sexual activity and marital status, psychiatric dysfunctions, hobbies and recreational activities, postoperative quality of life assessment) [34,35]. More recent studies conducted [15,37] identified other reasons for post-operative distress compared to older studies: the occurrence of complications, the duration of recovery, social support and family relationships, sexual activity, problems generated by the presence of the stoma, problems of social stigma, occupational recovery and potential decrease in income, and, last but not least, the fear of recurrence. These differences are probably generated by the fundamental change in the role of women in society—from a woman who was often a housewife, financially dependent on her husband, with recreational concerns and limited professional activity, to a financially independent woman, professionally active, with a supporting role in the family and society, more concerned about her own future from an oncological and socio-professional point of view. These aspects suggest the need for additional qualitative studies that better represent the realities of the modern era.

### 4.3. Expectations Regarding Treatment

Pelvectomies, particularly aggressive surgical interventions, are addressed to locally advanced primary and recurrent pelvic cancers, in which the effect of adjuvant therapies is questionable. As a result, the patient often sees this intervention as the only alternative to death and as a path to a cure. Even in a palliative context, pelvectomies are seen by patients as the only chance to get rid of a very troublesome symptom. In this context, patients are more inclined to accept the consequences of the surgical act that they would not otherwise accept.

*Patient 2: “I went to many doctors who all told me that they couldn’t do anything for me…that I came too late…I was very happy when I found the doctor who said he would operate on me. Everyone else sent me home to die…”*

*Patient 3: “I was bleeding so hard and I could barely stand. I smelled horrible, I had burns on my thighs from the feacal matter that was flowing uncontrollably…It was no life. I would have done anything to escape regardless of the risks.”*

Despite the explanations received pre-operatively, most subjects declare themselves surprised and stressed by the length of postoperative recovery and the complications that occurred [37]. Vera et al. [35] reported that 36.8% of patients considered postoperative recovery to be the most difficult thing they had faced.

### 4.4. Symptom Control

Usually, patients who are indicated for a pelvectomy present preoperatively a more or less important impairment of the general condition—usually due to neoplastic impregnation or complications of the disease (infection, bleeding, fistulas, pain syndrome). Preoperative symptomatology can also vary and affect various organs and systems. Exenterative surgery is usually followed by a period in which the patient reports a deterioration of the general condition, experiencing the common symptomatology after a major intervention (lethargy, physical asthenia, inappetence, psychological disorders including insomnia or anxiety-depressive episodes); however, these alterations are quite rapidly regressive. However, they can determine an impairment of QoL in these patients.

Postoperatively, patients may experience gastrointestinal symptoms such as abdominal distension accompanied by diffuse pain generated by the delay in the resumption of transit (ileus generated by intraoperative bowel manipulation and postoperative paresis of the intestinal mass) [14,42]. These symptoms are common to any abdominal surgery, are reversible, and do not represent alarm signs. However, certain postoperative gastrointestinal symptoms may signal the occurrence of complications—vomiting, inability to resume transit, exacerbation of abdominal pain syndrome, or occurrence of peritoneal irritation syndrome. These may mark the occurrence of digestive fistulas with secondary peritonitis, active bleeding, or patent intestinal occlusions. All of these require emergency surgical reintervention. Low/medium intensity gastrointestinal symptomatology may persist for even one year post-operatively [42].

Also, postoperatively, patients may present symptoms related to the genitourinary system. These, as a rule, appear in the late period and are difficulties in bladder mobility (due to damage to the hypogastric nerve plexuses during the intervention), leading to difficult evacuation. Pelvectomy may induce urinary incontinence in patients in whom the formation of an orthotopic neobladder was chosen [49]. Better QoL scores were reported in patients who underwent a double-barreled colonic conduit compared to those with urinary/fecal diversions [40]. As with digestive symptoms, some genitourinary symptoms may indicate a complication that will require reintervention—reduced or absent urine output on the urinary catheter, in parallel with increased drainage volume, may signal the development of a urethral/vesical fistula.

Pain is a common symptom in patients who are indicated for a pelvectomy and patients have high expectations of the ameliorating effect of the surgical intervention on pain. Pain syndrome has a strong effect on QoL and symptom control is the most frequent indication for palliative pelvectomy [43,47,48]. Guimaraes et al. [43] report pain relief and decreased need for analgesics (including major opioids) in all patients.

Of the patients interviewed, those who had severe symptoms prior to the intervention reported symptom relief. Hemorrhage, infection and digestive obstruction are resolved 100% by exenterative intervention if an R0 resection is possible. As a rule, the removal of the tumor also leads to eventual urinary decompression, which leads to improvement in renal function. Pain, which occurs due to compressive phenomena generated by the tumor or potential complications, is greatly reduced by the intervention. Of course, there is a degree of postoperative pain, but this follows the normal trajectory of operated patients, being generally controllable with usual analgesics. The need for chronic analgesic medication decreases after exenterations. In one of our patients, who presented with a complex ileovaginal fistula accompanied by chemical burns on the inner surface of the thighs (due to the alkalinity of the ileal contents), we observed another way in which pelvectomy can contribute to symptom control—by resection and closure of the ileal fistula, the skin on the thigh could be put to rest, which could subsequently heal properly (in the absence of chemical irritant).

One of the most important sources of stress for patients undergoing exenteration, contributing to problems with self-image perception, social interaction, and sexual dynamics, is the presence of one or more stomas and the practical difficulties of managing them [34,35,37]. Vera et al. [35] reported that one-third of patients found that adjusting to the realities of life with a stoma was the most difficult aspect of postoperative accommodation. However, after the initial period of adjustment and acceptance of the presence of the stoma, most of these drawbacks disappear [34,35,37]. Hawighorst-Knapstein et al. [46] reported that the level of distress caused by the presence of stomas is dependent on the number of stomas, with patients with total pelvicectomies and two stomas having worse physical and psychosocial function scores at 12 months compared with patients with posterior pelvicectomies (colostomy only) and patients with limited interventions to the organ of origin and no stomas. Similar results were also reported by Roos et al. [14]. Yet, Austin et al. [39] report comparable physical and psychosocial function scores in patients who underwent a total or posterior pelvicectomy.

### 4.5. Psychological Impact

The psychological impact of exenterative surgery is undeniable and patients report a series of emotional changes that follow a dynamic pattern throughout the different stages of diagnosis and treatment. Qualitative assessments have shown that the initial emotions upon learning the diagnosis, such as shock and fear, are quickly replaced by acceptance and hope for a possible cure presented by the proposed surgical intervention. Patients continue to experience episodes of sadness and depression in the preoperative period [34,35]. Immediate postoperative emotions are generally negative (depression and frustration caused by uncertainty about the future, including the perception of an increased degree of dependence on others, and changes in self-image perception) [34,35,37]. However, in evaluating the long-term psychological effects of pelvectomy, Vera et al. [2] reported that most patients considered the experience to be positive, improving their quality of life. Furthermore, they believed that the improvements would continue and expressed hope for the future. Authors such as Rezk et al. [42,46,56] have duplicated the qualitative assessment of the impact of exenteration on the patient’s psyche, with a quantitative assessment that produced similar results. These authors suggest that psychological changes (such as stress-induced ideation, depression) peaked in the immediate preoperative period and improved significantly one year after the intervention. Psychological changes are more pronounced in patients undergoing total pelvectomy compared to those undergoing hemipelvectomy [46,56].

The patients we interviewed also reported multiple emotional changes, demonstrating the myriad of consequences associated with pelvic exenteration, some of which the treating physician usually does not consider and does not discuss sufficiently with the patient and his family prior to the intervention. These changes suggest that psychological counseling should become an integral part of current oncological practice [57,58,59,60,61].

### 4.6. Strengths, Limitations, and Future Research Directions

This study offers one of the most comprehensive syntheses of data on quality of life following pelvic exenteration by combining both quantitative evidence from standardized outcome measures and qualitative data capturing lived patient experiences. The mixed-methods approach allows for a more integrated interpretation of postoperative recovery, reflecting not only functional outcomes but also patient meaning-making and coping processes—dimensions often absent from traditional surgical literature. Including data from both gynecological and colorectal cancer patients improves the generalizability of our findings across the most common indications for exenteration.

Nevertheless, several significant limitations must be acknowledged. The most significant is related to the data collection method for our qualitative interview-based study—we were able to interview very few patients and they cannot be considered statistically representative of the entire studied sample. The patients interviewed did not develop major postoperative complications and did not present an acute deterioration in their general condition after the operation. The qualitative component was based on only five patient interviews, a limitation that may affect generalizability. The aim of this study was to explore thematic insights and not to achieve statistical representation, and saturation was approached for key topics. These interviews, by not being limited by a rigid system of questions, but rather allowing the patient to freely express their feelings and opinions, with minimal guidance from the investigator, give us a picture of the reality these patients face and help us highlight the complexity of potential problems that most doctors do not think about, and certainly do not take into account when deciding on the therapeutic approach.

By far the most important limitation of the literature review is the heterogeneity in study design, follow-up duration, and QoL reporting, which makes it impossible for us to perform a truly meta-analytic synthesis or to adequately directly compare various studies. The use of varied and sometimes non-standardized instruments (e.g., EORTC QLQ-C30, CARES, SF-36, FACT-G) complicates cross-study interpretation. Only a few studies included baseline QoL assessments, making it difficult to gauge the actual impact of surgery over time. Moreover, a substantial proportion of the studies lacked detailed reporting of patient characteristics, such as the type of reconstruction or stoma configuration, which are known to influence postoperative body image and functionality. In the qualitative strand, variation in methodological transparency and rigor, as assessed through CASP scoring, may affect the transferability of findings.

Future research should aim to address these methodological gaps. There is a clear need for multicenter prospective studies using validated QoL instruments with consistent time points and longer follow-up periods (preferably beyond 24 months).

Development of a pelvic exenteration–specific QoL assessment module could improve both clinical decision-making and research comparability, similarly to tools developed in bladder cancer or other anatomically mutilating procedures [62,63,64]. A disease-specific QoL tool tailored to pelvic exenterations is needed. Based on the synthesis, such a module should assess stoma-related distress, sexual function stratified by gender, body image, psychosocial resilience, and role functioning. Integration with existing tools (e.g., EORTC modules) could improve feasibility. Stratification by surgical intent (curative vs. palliative), cancer type, and reconstruction method should also become standard reporting practice. Finally, combining patient-reported outcomes with in-depth qualitative exploration may provide the most complete insight into long-term adaptation and survivorship after this life-altering procedure.

## 5. Conclusions

This preliminary synthesis suggests that pelvic exenterations have a major impact on patients’ lives, not only physically, but also emotionally and socially. The quality of life after such an intervention depends on much more than the surgical or oncological results—it depends greatly on how patients adapt, how they feel about their own bodies, and how much support they receive from those around them. While some degree of recovery is possible over time, many patients continue to face challenges related to autonomy, intimacy, and identity. Postoperative adjustment is, however, favored by the fact that most patients view pelvectomy as the only alternative to imminent death and as a potential path to healing.

Standard questionnaires do not always capture all of these psycho-oncological aspects. Integrating patient perspectives—through qualitative data and patient-reported outcomes—is essential for a more complete understanding of postoperative recovery. Psychological support and individualized preoperative counseling should be considered core components of care, not optional additions. Future research would benefit from using consistent, patient-centered measures and including longer-term follow-up. Assessment tools for patients undergoing a pelvectomy that encompass all the specific modifications of the procedure are needed.

Recovery after pelvic exenteration is not only about clinical outcomes—it also involves how patients adapt, reconnect with themselves and others, and define life after treatment.

## Figures and Tables

**Table 1:** Main characteristics of included studies.

Study	Study Population	Study Design	Method of QoL Evaluation	Summary of Findings
Dempsey (1975) [34]	N = 16 gynecological cancers	Qualitative, Prospective	Qualitative interview	QoL and adaptation outcomes discussed
Vera (1981) [35]	N = 19 gynecological cancers	Qualitative, Retrospective	Qualitative interview	↓ Sexual function
Corney (1993) [36]	N = 8 gynecological cancers	Qualitative, Retrospective	Qualitative interview	↓ Sexual function
Carter (2004) [37]	N = 6 gynecological cancers	Qualitative, Retrospective	Qualitative interview	↓ Body image
Nelson (2021) [15]	N = 14 pelvic cancers	Qualitative, Prospective	Semi-structured interviews	↓ Sexual function
O’Dell (2023) [16]	N = 18 pelvic cancers	Qualitative, Prospective	Thematic analysis of patient interviews	Psychological distress
Forner (2011) [38]	N = 100 gynecological cancers	Quantitative, Retrospective	SF12	↓ Sexual function
Austin (2010) [39]	N = 37 CRC	Quantitative, Retrospective, case-controlled, cross-sectional	FACT-C SF36	↓ Overall QoL
Roos (2004) [14]	N = 32 gynecological cancers	Quantitative, Prospective	EORTC QLQ-C30 EORTC QLQ-OV28	↓ Overall QoL
Hsu (2014) [40]	N = 18 NS cancers	Quantitative, Retrospective, case-controlled	EORTC QLQ-C30	Persistent pain
Love (2013) [41]	N = 26 NS cancers	Retrospective	Sexual function questionnaire	Fatigue
Rezk (2012) [42]	N = 16 gynecological cancers	Quantitative, Prospective	EORTC QLQ-C30 EORTC-QLQ CR38, EORTC QLQBLM30 BFI, BPI-SF, IADL, CES-D, IES-R	↓ Overall QoL
Guimaraes (2011) [43]	N = 13 gynecological cancers	Quantitative, Retrospective	Unspecified symptoms scale	↓ Urinary function
Zoucas (2010) [3]	N = 85 CRC and gynecological cancers	Quantitative, Prospective	EORTC QLQ-C30	↓ Sexual function
Hawighorst (2004) [44]	N = 129 gynecological cancers undergoing PE (62 patients) or Wertheim procedure (67 patients)	Quantitative, Prospective	CARES, Preoperative Anxiety Scale	↓ Overall QoL
Mirhashemi (2002) [45]	N = 9 gynecological cancers	Quantitative, Retrospective	Symptoms scale	Persistent pain
Hawighorst-Knapstein (1997) [46]	N = 28 gynecological cancers	Quantitative, Prospective longitudinal	EORTC QLQ-C30, BIQ, CARES, Strauss—Appelt Body Image Score	↓ Overall QoL
Woodhouse (1995) [47]	N = 10, NS cancers	Quantitative, Retrospective	Symptoms scale	Psychological distress
Brophy (1994) [48]	N = 35, pelvic cancers	Quantitative, Retrospective	Symptoms scale	↓ Sexual function
Gleeson (1994) [30]	N = 14 patients with vaginal reconstruction after PE	Quantitative, Retrospective	Custom psychosexual questionnaire, semi-structured interviews	Stoma-related distress
Andersen (1983) [31]	N = 15 gynecological cancer	Quantitative, Retrospective	Sexual and psychosexual assessment though semi-structured interviews SCL90, BDI, KAS-R, MAT, DSFI, HTH, SAI	↓ Overall QoL
Radwan (2015) [13]	N = 110, CRC	Quantitative, Prospective	EORTC QLQ-C30	QoL and adaptation outcomes discussed
Young (2014) [2]	N = 182, CRC	Quantitative, Prospective	FACT-C, SF36, AuQoL, DT	Persistent pain
Beaton (2014) [7]	N = 31, CRC	Quantitative, Retrospective	FACT-C	↓ Overall QoL
Kato (2013) [49]	N = 49, gynecological cancer	Quantitative, Retrospective	UDI-6	↓ Sexual function
Alahmadi (2021) [6]	N = 710 patients post-PE	Quantitative, Retrospective	EORTC QLQ-C30	Persistent pain
Martinez (2018) [21]	N = 97 pelvic cancers	Quantitative, Prospective, Multicenter	EORTC QLQ-C30	Psychological distress
Dessole (2018) [5]	N = 96 recurrent gynecologic cancer	Quantitative, Prospective, Multicenter	EORTC QLQ-C30, DSFI	↓ Sexual function

Several included studies were based on small or very small cohorts (N < 10), which reflects the rarity of the procedure and the challenges in assembling large samples in this clinical population. Abbreviations: QoL—quality of life; N—sample size; CRC—colorectal cancer; PE—pelvic exenteration; SVQ—Sexual function-Vaginal changes questionnaire; EORTC QLQ-C30/ EORTC-QLQ CR38/ EORTC QLQBLM30/ EORTC QLQ-OV28—Quality of life questionnaire; BFI—Big Five Inventory questionnaire; BPI-SF—Brief pain inventory (short form); IADL—Instrumental Activities of Daily Living Scale questionnaire; CES-D—Center for Epidemiological Studies Depression Scale; IES-R—Revised Impact of Events Scale; SF-12—Short-form 12 health-survey questionnaire; FACT-C—Functional Assessment of Cancer Therapy-Colorectal questionnaire; SF36—36-Item Short Form Survey Instrument; CARES—Cancer Rehabilitation Evaluation System; SCL90—Symptom Checklist-90 questionnaire; BDI—Beck Depression Inventory questionnaire; KAS-R—Katz Social Adjustment Scale; MAT—Marital Adjustment Test; DSFI—Derogatis Sexual Functioning Inventory; SAI—Sexual Arousal Inventory; AuQoL—Australian Quality of Life Questionnaire; DT—Distress Thermometer Scale; HTH—Heterosexual Behavior Hierarchy; UDI-6—Urogenital Distress Inventory—6; ↓—decreased More details about studies findings can be found in the Supplementary Table S1.

## Data Availability

The datasets presented in this article are not readily available because the data supporting the findings of this study are not publicly available due to institutional restrictions and privacy issues.

## Supplementary Materials

The following supporting information can be downloaded at: https://www.mdpi.com/article/10.3390/jcm14186541/s1, Supplementary Table S1: Key-findings of included studies [2,3,5,6,7,13,14,15,16,21,30,31,34,35,36,37,38,39,40,41,42,43,44,45,46,47,48,49].

## Abbreviations

The following abbreviations are used in this manuscript:

AuQoL	Australian Quality of Life Questionnaire
BDI	Beck Depression Inventory
BFI	Big Five Inventory questionnaire
BPI-SF	Brief Pain Inventory-Short Form
CARES	Cancer Rehabilitation Evaluation System
CASP	Critical Appraisal Skills Programme
CES-D	Center for Epidemiological Studies Depression Scale
CRC	Colorectal Cancer
DSFI	Derogatis Sexual Functioning Inventory
DT	Distress Thermometer Scale
EORTC	European Organization for Research and Treatment of Cancer
EORTC QLQ-BLM30	European Organization for Research and Treatment of Cancer Quality of Life Questionnaire-Bladder Cancer Module
EORTC QLQ-C30	European Organization for Research and Treatment of Cancer Quality of Life Questionnaire-Core 30
EORTC QLQ-CR38	European Organization for Research and Treatment of Cancer Quality of Life Questionnaire-Colorectal Cancer Module
EORTC QLQ-OV28	European Organization for Research and Treatment of Cancer Quality of Life Questionnaire-Ovarian Cancer Module
FACT-C	Functional Assessment of Cancer Therapy-Colorectal questionnaire
HTH	Heterosexual Behavior Hierarchy
IADL	Instrumental Activities of Daily Living Scale questionnaire
IES-R	Revised Impact of Events Scale
IOB	Bucharest Institute of Oncology
KAS-R	Katz Social Adjustment Scale
MAT	Marital Adjustment Test
N	Sample size
NOS	Newcastle–Ottawa Scale
PE	Pelvic Exenteration
PRO	Patient-Reported Outcome
QLQ	Quality of Life Questionnaire
QoL	Quality of Life
SAI	State Anxiety Inventory
SCL90	Symptom Checklist-90 questionnaire
SF-12	Short Form-12 Health Survey Questionnaire
SF-36	36-Item Short Form Survey Instrument
SVQ	Sexual function-Vaginal changes questionnaire
UDI-6	Urogenital Distress Inventory-6
VRAM	Vertical Rectus Abdominis Myocutaneous
