# Supplementary material for: Living After Pelvic Exenteration: A Mixed-Methods Synthesis of Quality-of-Life Outcomes and Patient Perspectives
Source: J Clin Med. 2025 Sep 17;14(18):6541. doi: 10.3390/jcm14186541 (PMC12470396; doi:10.3390/jcm14186541)
Supplement: Supplementary file 1 [file jcm-14-06541-s001.zip › jcm-3820379-supplementary.pdf]

Supplementary Table S1. Key-findings of included studies.

Study	Key findings
Dempsey (1975) [34]	Return to occupational, social, and recreational activities post-exenteration
Vera (1981) [35]	Lasting impact on sexual and social function; overall perception of positive impact
Corney (1993) [36]	Sexual dysfunction reported in 66% of cases
Carter (2004) [37]	Impairment of body image and sexuality; gradual improvement over time
Nelson (2021) [15]	Major psychological impact, challenges in daily functioning and intimate relationships
O'Dell (2023) [16]	Persistent image and sexuality concerns; mixed experiences with recovery and support
Forner (2011) [38]	The study compared QoL in patients who had an ileal conduit and those who had an ileocecal pouch – similar results
Austin (2010) [39]	Comparable QoL outcomes in patients undergoing pelvectomy versus those undergoing anterior rectal resection or rectal amputation
Roos (2004) [14]	Women who have undergone a pelvectomy experience decreased physical and psychosocial function and a greater degree of financial hardship post-operatively. Acceptable quality of life post-PE; high scores on symptoms such as pain, fatigue, and insomnia; relatively good emotional adjustment.
Hsu (2014) [40]	The study compares results between two types of urinary diversions after PE. Postoperative improvements in health status and physical and social functioning
Love (2013) [41]	Only 14% of subjects report resuming sexual activity
Rezk (2012) [42]	QoL improved over time; satisfaction with care correlated with better QoL Respondents reported a decrease in QoL at 3 months but improvement at 12 months to baseline
Guimaraes (2011) [43]	All patients reported optimal control of the pain syndrome with a reduction in the need for analgesics.
Zoucas (2010) [3]	Initially decreased, QoL improved 16 months after surgery
Hawighorst (2004) [44]	Subjects with increased levels of preoperative anxiety experienced a greater decline in physical and psychosocial functions post-operatively. High anxiety level was correlated with a lack of information and dissatisfaction about physician-patient relationship
Mirhashemi (2002) [45]	Most women who had a pelvectomy followed by vaginal reconstruction and resumed sexual activity (78% of cases) are satisfied with the results.
Hawighorst-Knapstein (1997) [46]	Improved QoL over time but persistent image concerns<br>Number of stomas influences post-operative QoL
Woodhouse (1995) [47]	Approximately 80% of subjects report optimal symptom control.

Brophy (1994) [48]	88% - improved QoL<br>60% - pain control<br>50% - control of bleeding and fistulas
Gleeson (1994) [30]	High prevalence of psychosexual dysfunction and psychological distress Long-lasting impact on sexual function and persistent vulvar pain. Multiple flap-related complications. Appropriate counseling required
Andersen (1983) [31]	Severely impaired sexual function; relatively adequate psychological and social adjustment; moderate level of depression and stress.
Radwan (2015) [13]	Physical, mental and emotional functioning is initially diminished postoperatively, but these parameters become comparable at 3 months to those of patients with rectal amputations.
Young (2014) [2]	The initially low postoperative scores of patients with pelvectomies become similar to those of patients undergoing a smaller intervention after 9 months.
Beaton (2014) [7]	There is no direct relationship between preoperative nutritional status and postoperative QoL of subjects undergoing PE
Kato (2013) [49]	The rate of urinary complications increases initially but returns to baseline after 6 months
Alahmadi (2021) [6]	Elderly had better QoL but worse survival
Martinez (2018) [21]	QoL improved by 6–12 months; urostomy had less negative impact than colostomy; Elderly patients were the only ones with permanent decreased physical and social function.
Dessole (2018) [5]	Sexual function severely affected despite improved global QoL
